# Single-cell and spatial transcriptomics analysis of human adrenal aging

**DOI:** 10.1016/j.molmet.2024.101954

**Published:** 2024-05-06

**Authors:** Norifusa Iwahashi, Hironobu Umakoshi, Masamichi Fujita, Tazuru Fukumoto, Tatsuki Ogasawara, Maki Yokomoto-Umakoshi, Hiroki Kaneko, Hiroshi Nakao, Namiko Kawamura, Naohiro Uchida, Yayoi Matsuda, Ryuichi Sakamoto, Masahide Seki, Yutaka Suzuki, Kohta Nakatani, Yoshihiro Izumi, Takeshi Bamba, Yoshinao Oda, Yoshihiro Ogawa

**Affiliations:** 1 Department of Medicine and Bioregulatory Science, Graduate School of Medical Sciences, Kyushu University, Fukuoka, Japan; 2 Department of Computational Biology and Medical Sciences, Graduate School of Frontier Sciences, The University of Tokyo, Chiba, Japan; 3 Division of Metabolomics/Mass Spectrometry Center, Medical Research Center for High Depth Omics, Medical Institute of Bioregulation, Kyushu University, Fukuoka, Japan; 4 Department of Anatomic Pathology, Graduate School of Medical Sciences, Kyushu University, Fukuoka, Japan

**Keywords:** Adrenal cortex, Aging, Activator protein 1, Single-cell RNA sequencing, Spatial transcriptomics, WNT/β-catenin signaling

## Abstract

**Objective:**

The human adrenal cortex comprises three functionally and structurally distinct layers that produce layer-specific steroid hormones. With aging, the human adrenal cortex undergoes functional and structural alteration or “adrenal aging”, leading to the unbalanced production of steroid hormones. Given the marked species differences in adrenal biology, the underlying mechanisms of human adrenal aging have not been sufficiently studied. This study was designed to elucidate the mechanisms linking the functional and structural alterations of the human adrenal cortex.

**Methods:**

We conducted single-cell RNA sequencing and spatial transcriptomics analysis of the aged human adrenal cortex.

**Results:**

The data of this study suggest that the layer-specific alterations of multiple signaling pathways underlie the abnormal layered structure and layer-specific changes in steroidogenic cells. We also highlighted that macrophages mediate age-related adrenocortical cell inflammation and senescence.

**Conclusions:**

This study is the first detailed analysis of the aged human adrenal cortex at single-cell resolution and helps to elucidate the mechanism of human adrenal aging, thereby leading to a better understanding of the pathophysiology of age-related disorders associated with adrenal aging.

## Highlights

• The first single-cell and spatial transcriptomics studies of human adrenal aging.
• Abnormal layered structure of human adrenocortical tissues.
• Altered multiple signaling pathways underlies age-related adrenocortical changes.
• Macrophages mediate age-related adrenocortical cell inflammation and senescence.

## 1 Introduction

The human adrenal cortex is composed of three functionally and structurally distinct layers: zona glomerulosa (ZG), zona fasciculata (ZF), and zona reticularis (ZR), which produce a range of layer-specific steroid hormones, aldosterone, cortisol, and adrenal androgens, respectively. Adrenal steroid hormones, which are secreted in a coordinated manner, play critical roles in the regulation of various homeostatic mechanisms. However, with aging, the human adrenal cortex undergoes functional and structural alterations, thereby leading to the unbalanced production of adrenal steroid hormones, such as abnormal production of aldosterone and cortisol, and an apparent reduction of adrenal androgens [[1], [2], [3], [4]]. Even slight disturbances in adrenal steroid hormone production lead to the development of age-related disorders, such as hypertension, sarcopenia, and osteoporosis, thereby reducing both longevity and quality of life [1,5,6]. Thus, understanding the underlying mechanisms of age-related adrenocortical failure or “adrenal aging” could potentially contribute to extending the lifespan of individuals in the aging population.

The microstructure of the adrenal cortex is complex and heterogeneous, making a detailed layer-by-layer analysis technically difficult. Indeed, human adrenal aging has been mostly studied using histopathologic approaches, specifically by assessing changes in the layered structure using immunohistochemical staining with layer-specific steroidogenic enzymes. Accumulating evidence indicates that with aging, ZF expands, while ZG becomes discontinuous and shrinks, and ZR shrinks in size [1,2]. In addition to adrenocortical cells, the adrenal cortex is rich in immune cells, particularly macrophages [7,8]. These cells may play a role in the clearance of apoptotic adrenocortical cells; however, their involvement in the age-related alterations of the adrenal cortex has not been addressed. Furthermore, mice, which are often used as model organisms in aging research, differ from humans in their adrenocortical structure and function [9]. Most notably, they have only two adrenocortical layers (ZG and ZF); ZF has been shown to be formed centripetally from ZG along the WNT/β-catenin signaling gradient, with no functional ZR [[10], [11], [12]]. Therefore, the process of human adrenal aging is poorly understood.

Single-cell RNA sequencing (scRNA-seq) analysis can provide a complete picture of cellular functions by comprehensively profiling a substantial number of genes at the single-cell level. We recently performed scRNA-seq analysis of the adult human normal adrenal cortex and identified cell clusters corresponding to ZG, ZF, and ZR cells at single-cell resolution [13]. However, due to the loss of spatial information during scRNA-seq analysis, the relationship between functional and structural alterations in the human adrenal cortex has not been sufficiently studied.

Here, we conducted bulk RNA-seq, scRNA-seq, and spatial transcriptomics (ST) to elucidate the mechanisms linking the functional and structural alterations of the human adrenal cortex. We found that the layer-specific alteration of multiple signaling pathways underlies the abnormal layered structure in the human adrenal cortex with aging. Our data also suggest that macrophages are involved in age-related adrenocortical cell inflammation and senescence. This study provides the molecular and cellular basis for the unbalanced production of adrenal steroid hormones with aging, thereby offering a novel therapeutic strategy for preventing or treating age-related disorders associated with adrenal aging.

## 2 Materials and methods

### 2.1 Ethics

This study was approved by the institutional review board of Kyushu University (889-00). Informed consent was obtained from the patients upon admission to our hospital.

### 2.2 Sample preparation

All adult human adrenal samples were obtained during adrenalectomies at Kyushu University Hospital. The young sample was from the adrenal gland of a 27-year-old woman who underwent surgery for pheochromocytoma. The elderly samples were obtained from a 64-year-old man who underwent surgery for non-functioning adrenocortical adenoma and a 74-year-old man who underwent surgery for pheochromocytoma. After surgical removal, the adrenal glands were formalin-fixed, paraffin-embedded (FFPE), and stored at room temperature.

### 2.3 Library preparation and sequencing

scRNA-seq was performed using the fixed RNA Profiling (10x Genomics, Pleasanton, CA, USA). Two 25 μm FFPE tissue sections per sample were prepared and the tumor areas were trimmed off grossly. The sections were deparaffinized and dissociated into single cells according to the manufacturer's instructions (10x Genomics, CG000632: Isolation of Cells from FFPE Tissue Sections for Chromium Fixed RNA Profiling). The prepared single cells were processed using Chromium Fixed RNA Profiling Reagent Kits (10X Genomics, catalog #1000474, #1000422, #1000251) and libraries were constructed according to the manufacturer's instructions (10x Genomics, CG000477: Chromium Fixed RNA Profiling Reagent Kits). The libraries were sequenced using a NovaSeq6000 (Illumina). Mapping and counting were performed using Cell Ranger (version 7.1.0) with the reference genome GRCh38-2020-A.

ST was performed using the Visium Spatial Gene Expression for FFPE (10x Genomics). A 6.5 mm square, 5 μm thick FFPE tissue section was prepared for each sample. The sections were placed in each capture area of the gene expression slide and processed using Visium Spatial Gene Expression Reagent Kits for FFPE (10X Genomics, catalog #1000185, #1000362, and #1000364) according to the manufacturer's instructions. Libraries were sequenced using MGI DNBSEQ-G400. Mapping to the reference genome GRCh38-2020-A, counting, and integration of expression data with hematoxylin–eosin (HE) staining images were performed using Space Ranger (version 1.3.1).

### 2.4 scRNA-seq and ST analysis

#### 2.4.1 Quality control (QC)

For QC of the scRNA-seq data, ambient RNA was first decontaminated using the decontX function in the R package celda (version 1.16.1) [14]. Doublets were removed using the R package scDblfinder (version 1.14.0) with default settings [15]. Cells with more than 8,000 expressed genes were also considered doublets and removed. Cells with fewer than 500 expressed genes were considered low-quality/dead and removed.

For QC of the ST data, spots with fewer than 500 expressed genes were considered low quality and removed. The Loupe Browser (10x Genomics) was used to identify spots located outside the adrenal gland by matching the location of the spots with HE-stained images, and these spots were removed from the analysis.

#### 2.4.2 Normalization, batch correction, and dimensional reduction

After QC, scRNA-seq and ST data were processed using the R package Seurat (version 4.3.0) [16]. The unique molecular identifier (UMI) count data were normalized using the regularized negative binomial regression method implemented in the SCTransform function. Principal component (PC) analysis using the RunPCA function was performed on the top 3000 highly variable genes.

Batch-corrected PC embeddings were calculated using the R package harmony (version 0.1.1) [17]. Uniform manifold approximation and projection (UMAP) was performed using the RunUMAP function with the top 30 batch-corrected PC embeddings [18]. Comparisons of the compositional proportions of cell types were performed using the Fisher's exact test. Benjamini–Hochberg correction was used to correct for multiple comparisons.

#### 2.4.3 Deconvolution analysis

ST data were deconvoluted using the scRNA-seq data with the same samples using the create.RCTD and run.RCTD functions of the R package spacexr (version 2.0.6) [19]. The “doublet_mode” parameter of the run.RCTD function was set to full.

#### 2.4.4 Differential expression analysis

Differential expression analysis was performed using the FindMarkers or FindAllMarkers function with the Wilcoxon rank-sum test. P-values were adjusted for multiple testing using the Benjamini–Hochberg correction. Differentially expressed genes (DEGs) were defined as those with a log fold change >0.25 and an adjusted P-value <0.05.

#### 2.4.5 Enrichment analysis

Gene set enrichment analysis (GSEA) was performed using the R package fgsea (version 1.24.0) [20], and hallmark gene sets from the Molecular Signatures Database and SenMayo, a gene set of the senescence-associated pathway, were examined [21,22].

Single-sample GSEA was performed using the R package escape (version 1.10.0), and enrichment scores for each gene set were calculated per cell and spot [23].

#### 2.4.6 Gene regulatory network (GRN) inference analysis

GRN was inferred using SCENIC (version 1.3.1) and pySCENIC (version 0.12.1) [24]. ZG, ZF, and ZR cells were extracted from the scRNA-seq raw count data, log2-normalized, and used as the input for pySCENIC. The transcription factor (TF) list and TF binding database were obtained from the cisTarget resources (https://resources.aertslab.org/cistarget/). The TF list was obtained from https://resources.aertslab.org/cistarget/tf_lists/allTFs_hg38.txt, and the TF binding databases from https://resources.aertslab.org/cistarget/databases/homo_sapiens/hg38/refseq_r80/mc9nr/gene_based/hg38__refseq-r80__10kb_up_and_ down_tss.mc9nr.genes_vs_motifs.rankings.feather and https://resources.aertslab.org/cistarget/databases/homo_sapiens/hg38/refseq_r80/mc9nr/gene_based/hg38_refseq-r80_500bp_up_and_100bp_down_tss.mc9nr.genes_vs_motifs.rankings.feather. Regulon activity score matrices calculated using pySCENIC were loaded onto R using SCENIC. The calcRSS function was used to calculate the regulon specificity score (RSS) of each cell type.

#### 2.4.7 Trajectory analysis

ZG, ZF, and ZR cells of the young sample were selected from the scRNA-seq data and dimensionally reduced on a diffusion map using the DiffusionMap function of the R package destiny (version 3.14.0) [25]. The top 30 PCs were used as the input for the DiffusionMap function. The differentiation trajectory from ZG to ZR was inferred in the diffusion map space using the slingshot function of the R package slingshot (version 2.8.0), and the pseudotime of each cell was calculated [26].

#### 2.4.8 *In silico* gene perturbation analysis

*In silico* gene perturbation analysis was performed using the python package CellOracle (version 0.12.0) [27]. The GRN used in CellOracle was the built-in hg38_gimmemotifsv5_fpr2. The anndata was created based on the count data focused on the top 3000 variable genes, the diffusion component information, and the pseudotime of each cell estimated by trajectory analysis, and input into CellOracle using the import_anndata_as_raw_count function. GRN preprocessing, perturbation, and perturbation score calculation were performed according to the CellOracle tutorial (https://morris-lab.github.io/CellOracle.documentation/tutorials/index.html). Shifts in cell identity due to perturbation were simulated by setting the expression of each transcription factor to zero or twice the maximum expression.

#### 2.4.9 Assortativity analysis

The assortativity scores of adrenocortical layers, which represent the continuity of layer structure, were calculated using the RunLabelAssortativityTest function of the R package semla (version 1.0.0) [28]. First, four regions of interest (ROIs) (Supplementary Fig. 6) for each sample, which ranged from capsule to medulla, 0.5 mm wide, were arbitrarily selected using the addSpatialTrajectory function of the R package SPATA2 (version 2.0.1) [29]. The assortativity scores of the ROIs were compared using the Wilcoxon rank-sum test. Statistical significance was set at P-value <0.05.

#### 2.4.10 Cell–cell interaction analysis

Cell–cell interaction analysis was performed using the R package LIANA (version 0.1.12) [30]. The scRNA-seq data were divided into young and elderly, and ligand–receptor interactions were estimated between each cell type according to the tutorial of LIANA (https://saezlab.github.io/liana/articles/liana_tutorial.html). Interactions with a P-value less than 0.05, as determined by the CellPhoneDB algorithm, were considered significant. The strength of the ligand–receptor interaction (LR score) was calculated by the SingleCellSignalR algorithm. The R package CrossTalkeR (version 1.3.5) [31] was used to compare the changes in interactions between young and elderly. Interactions with differences in LR score greater than 0.7 were considered as significantly changed.

### 2.5 Genotype-tissue expression (GTEx) data analysis

RNA-seq data and sample information of the normal adrenal cortex from the GTEx were obtained using R package recount3 (version 1.6.0) [32]. The young group was defined as those younger than 60 years old, and the elderly group was defined as those older than 60 years old. Samples with low RNA quality (RNA integrity number <6) were excluded.

Gene expression data were used as inputs for the R package edgeR (version 3.40.2) [33]. Genes with low expression levels were excluded using the filterByExpr function. Gene counts were normalized by applying the trimmed mean of M-values normalization method using the calcNormFactors and cpm functions. DEGs were detected using the glmFit and glmLRT functions. The Benjamini–Hochberg method was used to correct for multiple comparisons. DEGs were defined as genes with an absolute value of log2 fold-change greater than 0.25 and an adjusted P-value of less than 0.05. GSEA was performed using the R package fgsea (version 1.24.0) [20], and hallmark pathway gene sets were examined. Gene sets with adjusted P-values of less than 0.05 were defined as significant.

### 2.6 Immunohistochemical (IHC) analysis

IHC was performed on adrenal tissue associated with pheochromocytoma or nonfunctioning tumor in 3 young and 2 elderly patients, in addition to the 3 patients for whom scRNA-seq/ST analysis was performed. The patient list is summarized in Supplementary Table 17. IHC of CD68 (RRID AB_2074844) as macrophage marker and p21 (RRID AB_395318) as senescent cell marker were performed (Supplementary Table 10). In each tissue section, three 750 μm-square ROIs were randomly selected, each containing mostly adrenal cortex, and the percentage of positively-stained cells was calculated using the positive cell detection function of QuPath [34].

### 2.7 Steroid profiling

#### 2.7.1 Patients

A total of 87 patients with non-functioning adrenocortical tumors admitted to our endocrine unit between January 2013 and September 2021 were included in this study. Patients with aldosterone-producing adenomas, cortisol-producing adenomas, adrenocortical carcinomas, and pheochromocytomas were excluded based on clinical data, imaging features, and postoperative histopathologic data. Patients with midnight cortisol levels ≥5 μg/dL or cortisol levels after a 1 mg dexamethasone suppression test (DST-cortisol) ≥1.8 μg/dL were considered to have autonomous cortisol production and excluded. Steroid profiling was performed on a total of 29 patients, defined as young (<50 years old) and elderly (≥70 years old).

Plasma adrenocorticotropic hormone (ACTH), serum cortisol, and serum dehydroepiandrosterone-sulfate (DHEA-S) levels were evaluated in morning blood samples. Midnight serum cortisol levels were evaluated using a 23:00 blood sample. The 24-h urinary free cortisol and DST-cortisol levels were also evaluated. These baseline characteristics, including age, were tested for differences between young and elderly by the Wilcoxon-rank sum test. Fisher's exact test was used to determine differences in sex ratio.

#### 2.7.2 Steroid profiling

Peripheral venous blood was collected the next morning after overnight fasting, and plasma was separated from whole blood by centrifugation and stored in a deep freezer (−80 °C) until analysis. Plasma steroid metabolites were measured as previously described [35,36]. The steroid metabolites listed in Supplementary Table 21 were evaluated.

#### 2.7.3 Analysis of plasma steroid profiles

Downstream analysis of the metabolome data was performed using MetaboAnalyst 5.0 [37]. The raw steroid profile data were transformed using the normalization functions (log transformation and autoscaling). Three (0.7%) missing values were detected and were replaced with 1/5 of the min-positive values of their corresponding variables.

Student's t-test was performed to detect differences in metabolite concentrations. P-values were adjusted for multiple testing using the Benjamini–Hochberg correction. We defined log2 fold-change >0.25 and adjusted P-value <0.05.

Supervised partial least squares-discriminant analysis (PLS-DA) was performed to determine whether steroid profiles distinguished the young and elderly groups. Variable importance in the projection (VIP) score analysis was performed to rank the overall contribution of each variable to the PLS-DA model, and variables with VIP scores >1 were considered significant.

## 3 Results

### 3.1 Bulk RNA-seq analysis of aged human adrenal cortex

**Figure 1:**
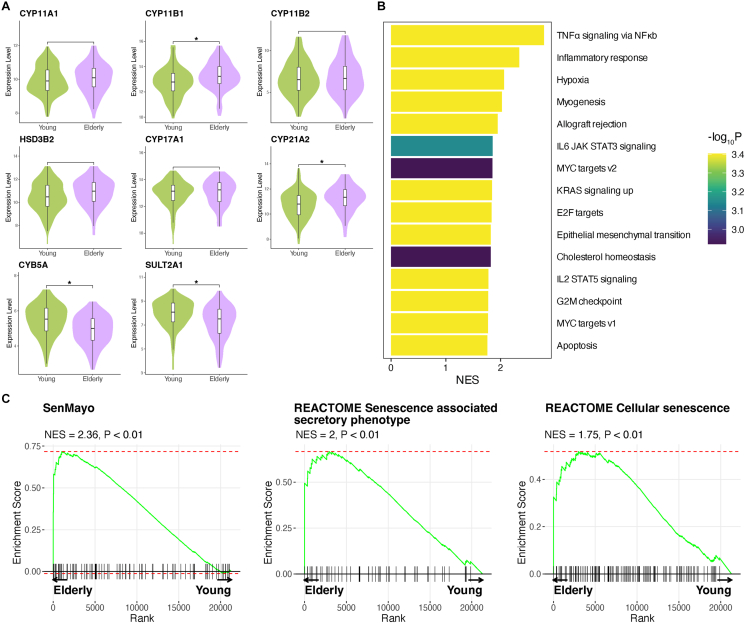
**Bulk RNA-seq analysis of the young and elderly adrenal cortex**. **A** Violin plots showing gene expression of steroidogenic enzymes. Asterisks indicate significant expression changes. **B** Bar plot showing the result of gene set enrichment analysis (GSEA) comparing between the young and elderly. Selected gene sets are shown. Gene sets with positive normalized enrichment scores (NES) indicate the positive enrichment of genes in the elderly. **C** GSEA results for senescence-associated gene sets.

To elucidate the age-related alterations in the human adrenal cortex, we performed bulk RNA-seq analysis of normal adrenal cortices (104 young males, 59 young females, 36 elderly males, and 33 elderly females), for which the data were obtained from the GTEx project [38] (Supplementary Table 1). Differential expression analysis was performed to compare gene expression profiles between young and elderly. Regarding steroidogenic genes, the expression of *CYP11B1* and *CYP21A2*, which are required for cortisol synthesis, was upregulated in the elderly. In contrast, the expression of *CYB5A* and *SULT2A1*, which are required for androgen synthesis, was downregulated (Figure 1A, Supplementary Fig. 1, Supplementary Table 2).

GSEA using the Molecular Signatures Database hallmark gene sets showed the expression of TNFα signaling and inflammatory response genes was upregulated in the adrenal cortex of the elderly (Figure 1B, Supplementary Table 3). Given that these molecular pathways are related to the hallmarks of cellular senescence [39], GSEA was performed on the adrenal cortex of the elderly using the senescence-associated gene sets. In this study, significant enrichment was observed in SenMayo [21] and two senescence-associated gene sets in reactome [40], suggesting the increased cellular senescence in the adrenal cortex of the elderly (Figure 1C, Supplementary Table 3). GSEA was also performed for WNT/β-catenin and cAMP/PKA signaling, both of which play critical roles in adrenocortical cell differentiation in mice [11,12,41], and no significant enrichment were found between the young and elderly (Supplementary Fig. 1).

### 3.2 scRNA-seq analysis of aged human adrenal cortex

**Figure 2:**
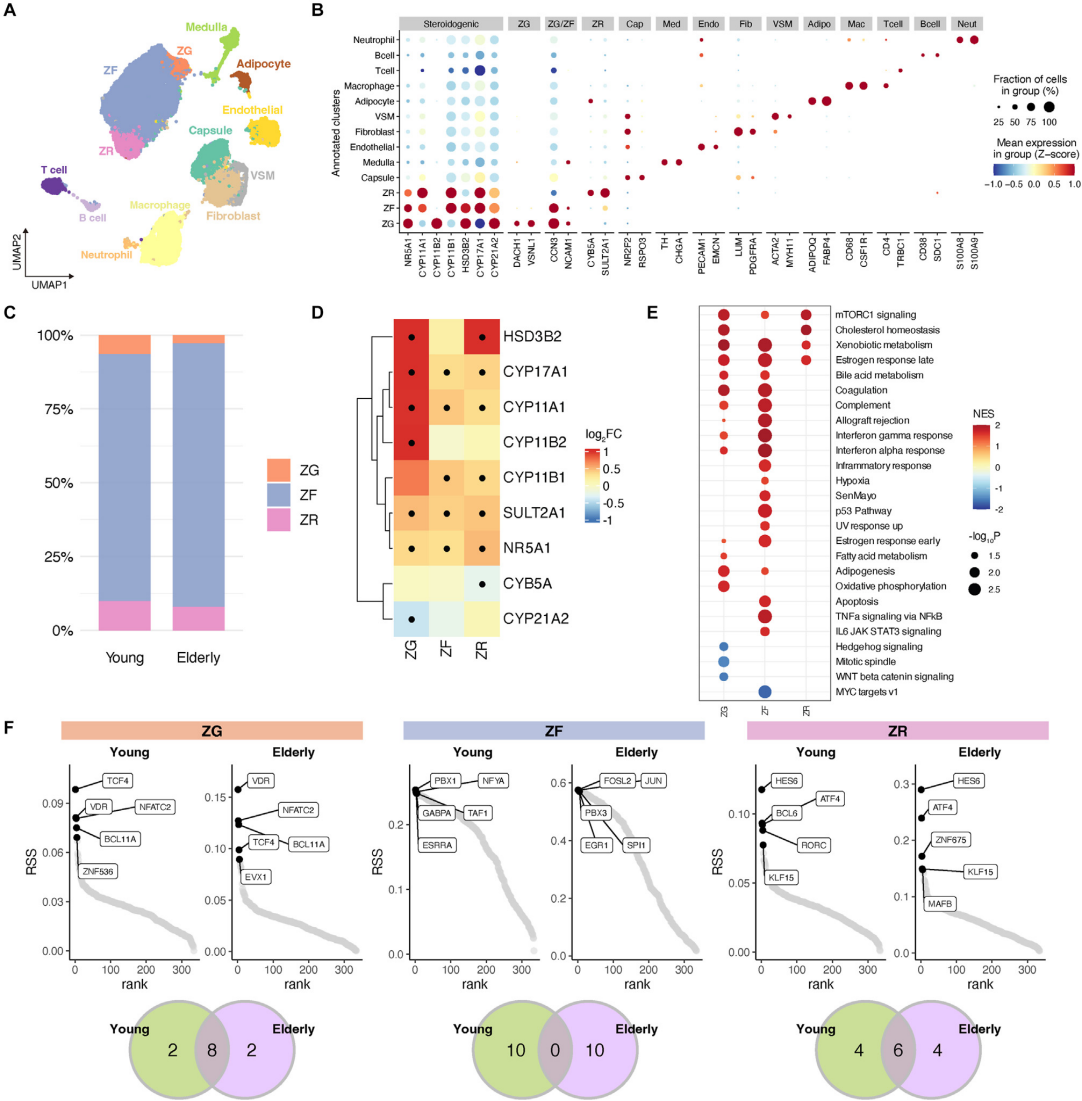
**scRNA-seq analysis of the young and elderly adrenal cortex**. **A** UMAP plot of scRNA-seq data colored by cell type annotation. **B** Dot plot showing the expression of marker genes in the annotated clusters of scRNA-seq data. **C** Bar plot showing the composition of ZG, ZF, and ZR cells in the young and elderly. **D** Heatmap showing the expression of steroidogenic enzyme genes in each adrenocortical layer compared between the young and elderly. Genes with positive values indicate upregulation in the elderly. **E** Dot plot showing the result of gene set enrichment analysis comparing the young and elderly in each adrenocortical layer of scRNA-seq data. Gene sets with significant enrichment are shown. Gene sets with positive normalized enrichment scores (NES) indicated the positive enrichment in the elderly. **F** TFs ranked by regulon specificity score (RSS), showing the top five TFs with the highest RSS per cell type. The bottom row is a Venn diagram showing the number of overlaps between the young and elderly in the RSS Top 10 TFs.

To elucidate the age-related alterations in human adrenocortical tissues at single-cell resolution, we performed scRNA-seq analysis on non-tumorous adrenal tissues obtained from three patients (a 27-year-old woman, and 64- and 74-year-old men) with pheochromocytoma or non-functioning adrenocortical tumor. The scRNA-seq data of 35,736 cells were clustered to identify adrenocortical cells containing ZG, ZF, and ZR, based on marker gene expression [13] (Figure 2A and B, Supplementary Fig. 2, Supplementary Tables 4–7). The proportions of the ZG and ZR cells in adrenocortical cells were lower in the elderly than in the young (Figure 2C).

Differential expression analysis and GSEA were performed to compare gene expression between the young and elderly in ZG, ZF, and ZR cells. Regarding the steroidogenic genes, the expression of *HSD3B2* and *CYP17A1* in ZG cells, *CYP17A1* and *CYP11B1* in ZF cells, and *HSD3B2* in ZR cells was upregulated in the elderly compared to that in the young (Figure 2D, Supplementary Table 8). GSEA revealed that the gene set of mTORC1 signaling, which is known to be activated in various tissues with aging [42,43], shows positive enrichment in ZG, ZF, and ZR cells of the elderly (Figure 2E, Supplementary Table 9). The gene set of WNT/β-catenin signaling, which plays a critical role in the maintenance of ZG in mice [11,41], showed negative enrichment in ZG cells of the elderly (Figure 2E, Supplementary Table 9). In ZF cells of the elderly, multiple gene sets showed positive enrichment, including those related to inflammation and immunity, such as “TNFα signaling via NFκB” and “inflammatory response”, as well as those associated with cell differentiation and proliferation, such as the “p53 pathway”, “hypoxia”, and “apoptosis”. In addition, the senescence-associated “SenMayo” gene set [21], was also enriched. By contrast, no such gene sets were enriched in ZG and ZR cells of the elderly. There was no significant enrichment in the gene set of cAMP/PKA signaling in ZG, ZF, and ZR cells.

Next, we performed a GRN analysis using SCENIC [24] to investigate the transcription factors that cause gene expression changes with aging. In both ZG and ZR cells, most of the activated transcription factors were shared between the young and elderly, while in ZF cells, they differed between the young and elderly. In ZF cells of the elderly, *JUN* and *FOSL2*, members of activator protein 1 (AP-1), a transcription factor known to regulate transcriptional responses to various stimuli such as stress, inflammation, and aging [44], were activated (Figure 2F).

### 3.3 *In silico* perturbation analysis

**Figure 3:**
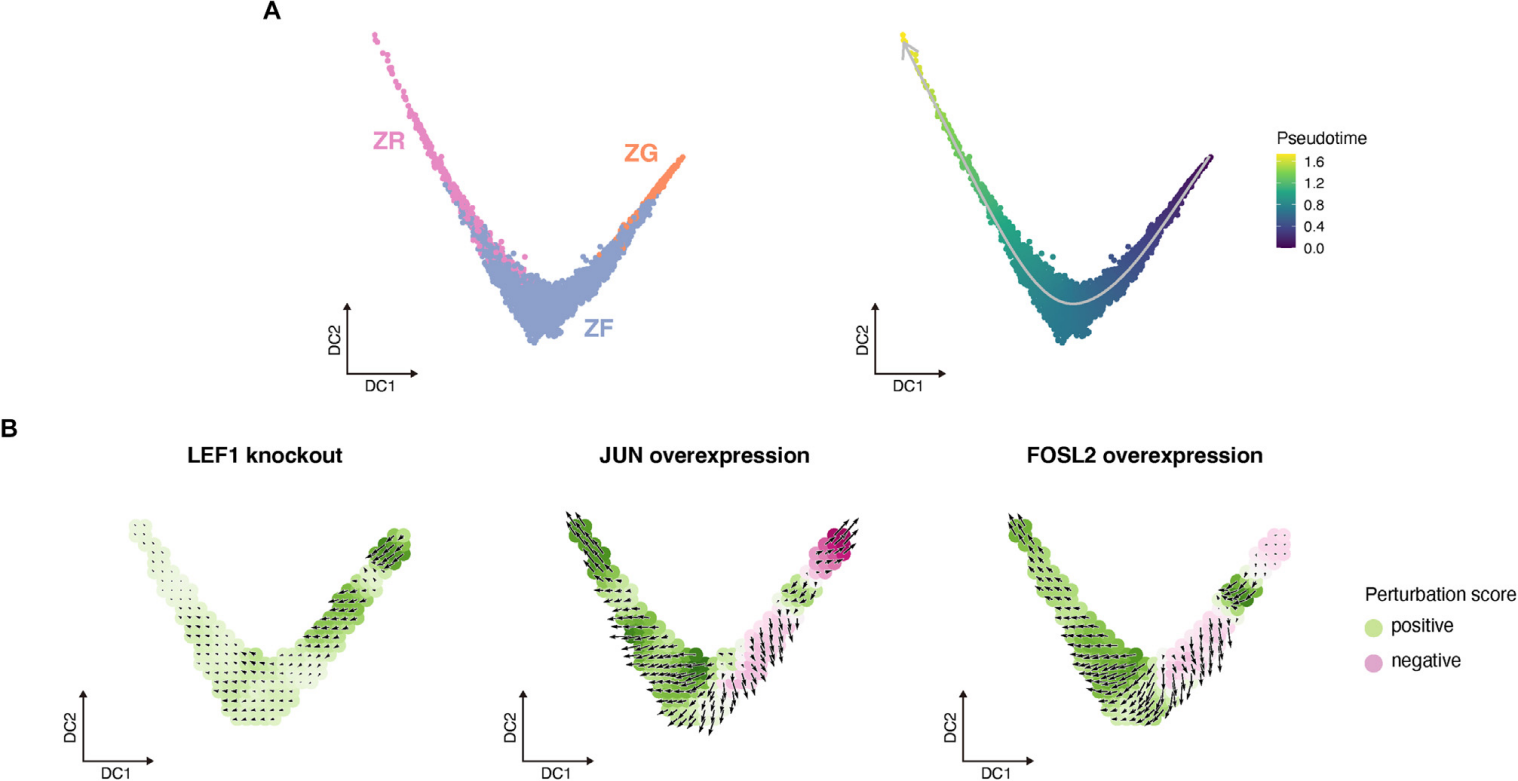
**Effect of perturbation of WNT/β-catenin and AP-1 family members on adrenocortical cell differentiation**. **A** Trajectory analysis inferring the adrenocortical cell differentiation. Adrenocortical cells of the young were dimensionally reduced onto a diffusion map. Left panel shows cell type. Right panel shows the inferred trajectory (gray arrows) and pseudotime of each cell. **B** Effects of *LEF1* knockout and *JUN* and *FOSL2* overexpression on cell differentiation. Black arrows represent shifts in cell identity owing to perturbations. The color of each cell indicates the perturbation score (PS). Negative PS (red) indicates that TF perturbation inhibited differentiation. Positive PS (green) indicates that TF perturbation promoted differentiation.

Given that the WNT/β-catenin signaling is attenuated in ZG cells and that the AP-1 family members are activated in ZF cells with aging, we performed *in silico* perturbation analysis to investigate the effect of these age-related changes on adrenocortical cell differentiation. In preparation for this analysis, trajectory analysis was performed on adrenocortical cells of the young to infer the differentiation trajectory. As a result, a trajectory of differentiation from ZG through ZF to ZR cells was inferred (Figure 3A). Along the trajectory, the WNT/β-catenin signaling showed a significant negative gradient (r = −0.38, P < 0.01), while cAMP/PKA signaling had a weak but positive gradient (r = 0.15, P < 0.01) (Supplementary Fig. 3). These observations are consistent with those previously reported in experimental mouse studies and in human adrenocortical scRNA-seq studies [10,13,41,45].

Next, we simulated whether cell differentiation is promoted or inhibited in adrenocortical cells of the young through the attenuation of WNT/β-catenin signaling or activation of the AP-1 family members. Knockout of *LEF1*, a target gene for WNT/β-catenin signaling, promoted differentiation of ZG cells towards ZF cells (Figure 3B). On the other hand, overexpression of *JUN* and *FOSL2*, which are activated in ZF cells of the elderly, suppressed ZF cell differentiation (Figure 3B).

### 3.4 ST analysis of aged human adrenal cortex

**Figure 4:**
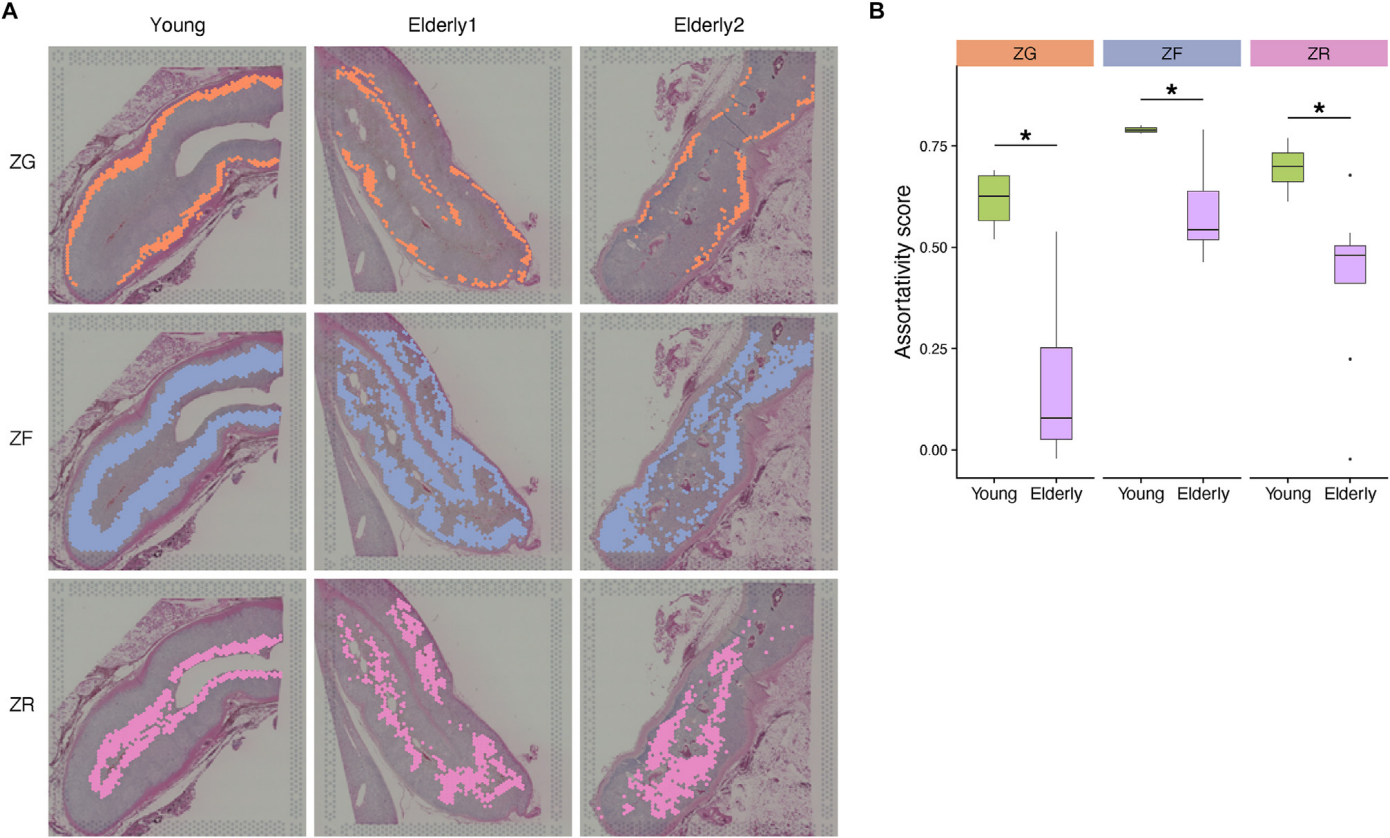
**ST analysis of the young and elderly adrenal cortex**. **A** Split view of the spatial location of ST spots annotated as ZG, ZF, and ZR. **B** Box plot showing the result of the assessment of continuity in the adrenocortical layers using ST data. Higher assortativity scores indicate that the layers are more continuous. Asterisks indicate significant differences (P value < 0.05) according to the Wilcoxon rank-sum test.

To verify the relationship between the functional and structural alterations in the adrenal cortex with aging, we performed ST analysis on the same adrenocortical tissues as those used for scRNA-seq analysis. The ST data from 7,022 spots were clustered to identify adrenocortical cells containing ZG, ZF, and ZR, based on the marker gene expression [13] (Figure 4A, Supplementary Figs. 4–6, Supplementary Tables 10–14). As expected, each adrenocortical layer in the young was continuous, with ZG, ZF, and ZR being clearly separated. In contrast, the layered structure was disrupted in the elderly (Figure 4A). To quantitatively assess the continuity of each adrenocortical layer, an assortativity analysis was performed to score the connectivity between spots. All three adrenocortical layers had lower assortativity scores in the elderly than in the young (Figure 4B).

The proportion of ZG in the adrenal cortex was lower in the elderly than in the young (Supplementary Fig. 6). The expression of *LEF1*, and *WNT4*, the major WNT ligand in the adrenal cortex [41], was downregulated in ZG of the elderly (Supplementary Tables 15 and 16). There were no significant differences in the proportions of ZF and ZR in the adrenal cortex between the young and elderly (Supplementary Fig. 6). The expression of several AP-1 family members, such as *JUN* and *FOS*, was upregulated in ZF (Supplementary Tables 15 and 16). In this study, the results obtained from the ST analysis are generally consistent with those by the scRNA-seq analysis regarding the changes in ZG and ZF.

### 3.5 Characterization of macrophages in aged human adrenal cortex

**Figure 5:**
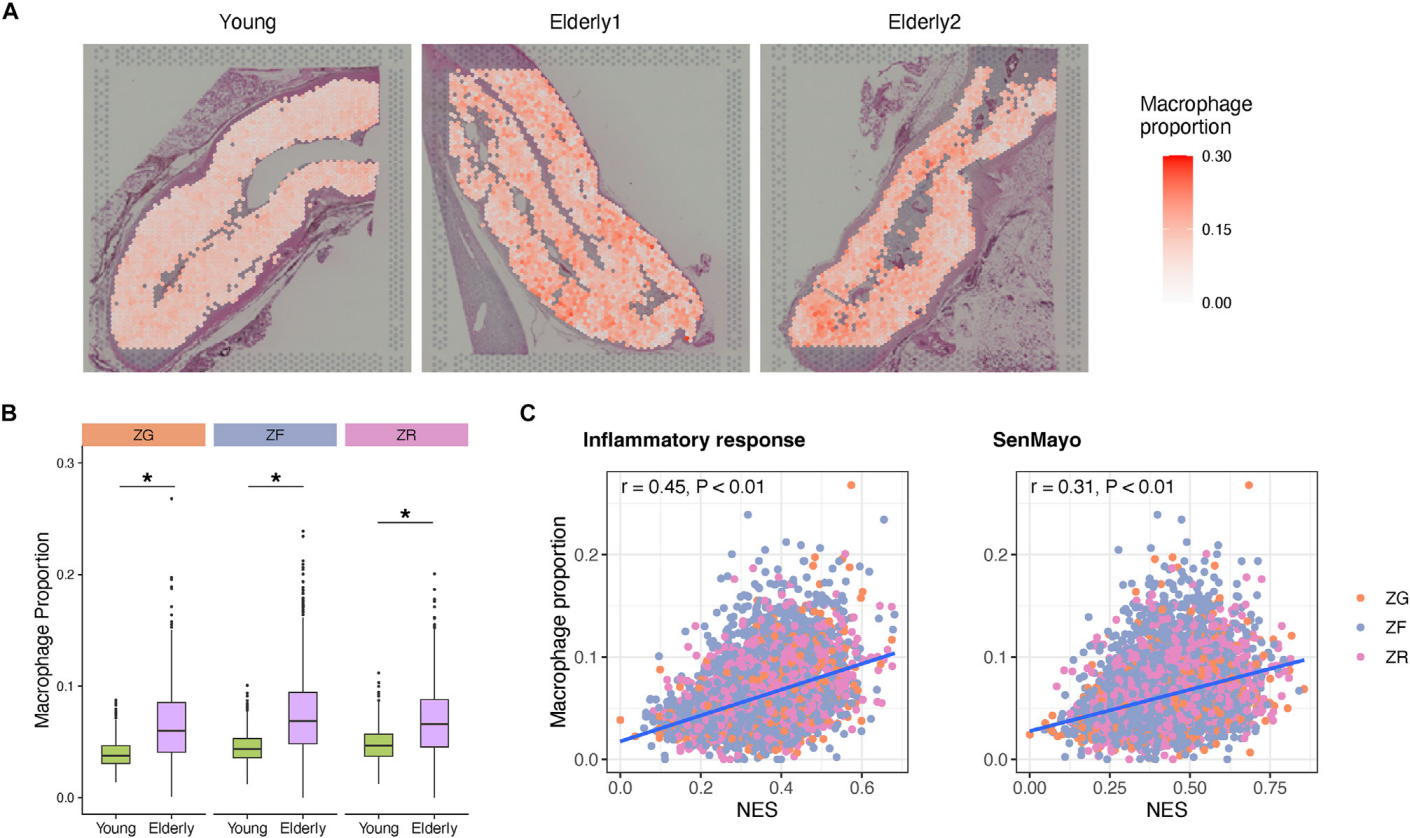
**Estimated localization of macrophages in the young and elderly adrenal cortex**. **A** Split view of the spatial distribution of the estimated macrophage proportions in ZG, ZF, and ZR. **B** Box plot showing the estimated macrophage proportions. Asterisks indicate significant differences (P value < 0.05) according to the Wilcoxon rank-sum test. **C** Dot plot showing the correlation between the estimated macrophage proportions and the normalized enrichment score of the hallmark inflammatory response and SenMayo gene sets. Dot colors represent the annotated cell types and the blue line represents the linear regression line.

Because the adrenal cortex is rich in immune cells, particularly macrophages, in healthy subjects [7,8], we addressed the role of macrophages in the human adrenal cortex with aging. The proportion of macrophages in each spot was estimated by deconvolution analysis (Figure 5A and B). In this study, with all the three cases examined; one from the young and two from the elderly, the proportion of macrophages showed a significant positive correlation with the enrichment score of Inflammatory (r = 0.45, P < 0.01) and SenMayo (r = 0.31, P < 0.01) gene sets (Figure 5C), suggesting that macrophages exist in close proximity to adrenocortical cells undergoing cellular inflammation and senescence. Immunohistochemical staining evaluation also confirmed that the percentage of positive cells for CD68 (macrophage marker) [46] and p21 (senescence marker) [47] were higher in the elderly than in the young (Supplementary Fig. 7, Supplementary Table 17).

Between the young and elderly, the proportion of macrophages was higher in the elderly than that in the young for all three adrenocortical layers (Figure 5B). In the young, the proportion of macrophages was the highest in ZR, while in the elderly, the proportion was the highest in ZF. Therefore, we examined the interactions of macrophages and ZF cells using the scRNA-seq data. In ZF of the elderly, the interaction mediated by LRP1, an “Eat me signal” molecule, was enhanced, while those mediated by B2M, a “Don't eat me signal” molecule, was attenuated relative to ZF of the young [48] (Supplementary Fig. 8, Supplementary Table 18).

To further compare macrophage gene expression between the young and elderly, GSEA was performed using the scRNA-seq data. In this study, the “Adipogenesis” and “Cholesterol homeostasis” gene sets were enriched in the elderly. In the elderly, the M1 macrophage gene set [49] showed significantly negative enrichment, whereas the M2 macrophage gene set [49] showed positive enrichment, with no statistical significance (Supplementary Fig. 8, Supplementary Table 19).

### 3.6 Characterization of plasma profiles of steroid metabolites with aging

To gain insight into the impact of aged adrenal cortical alteration on steroid hormone synthesis, we characterized the plasma profiles of 15 steroid metabolites in 29 hospitalized patients (13 young and 16 elderly) diagnosed with non-functioning adrenocortical tumors. Baseline characteristics of the patients are presented in Supplementary Table 20. In this study, there were no significant differences in morning ACTH or cortisol levels between the young and elderly. Plasma cortisol levels after the 1 mg dexamethasone suppression test and at midnight were higher in the elderly than those in the young (Supplementary Table 20).

**Figure 6:**
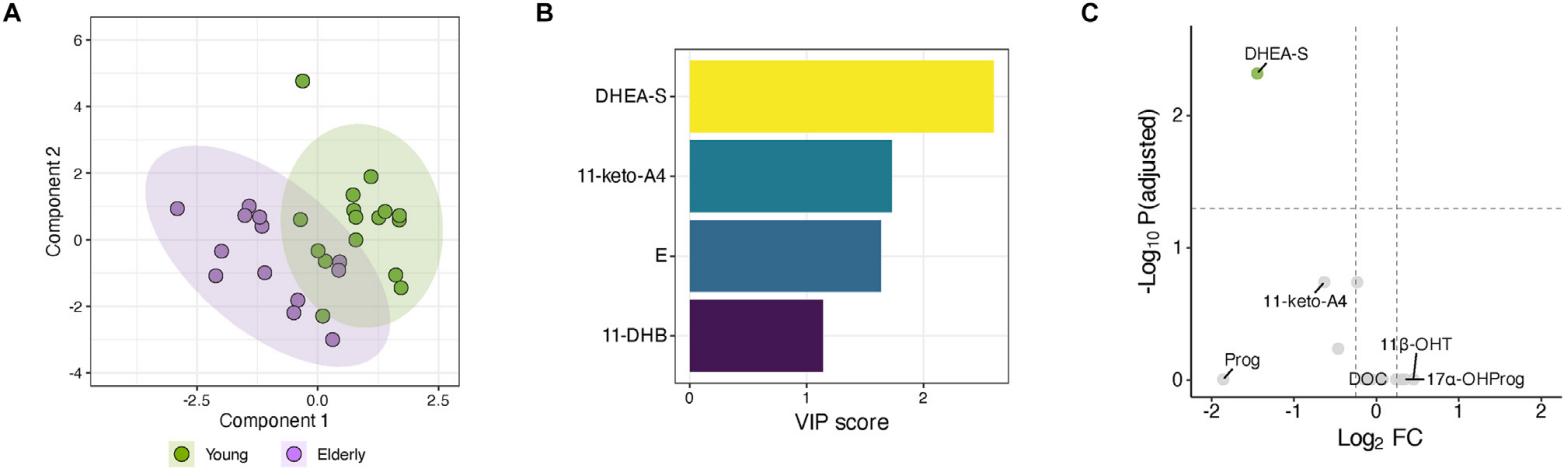
**Plasma steroid profiling of the young and elderly**. **A** Scores plot showing the result of partial least squares-discriminant analysis (PLS-DA) between young and elderly. Shaded circles indicate 95% confidence intervals. Dots represent individual samples. **B** Variable importance in projection (VIP) scores of component 1 of the PLS-DA. Metabolites with a VIP score of 1 or higher are shown. **C** Volcano plot showing the differences in the concentrations of metabolites between young and elderly. The vertical and horizontal lines indicate the log2 fold-change (log2FC) threshold of 0.25 and the adjusted P-value threshold of 0.05, respectively. Metabolites with positive log2FC indicate the higher concentrations in the elderly than in the young. The top three metabolites with the largest fold changes are highlighted.

PLS-DA was performed on the plasma steroid profile, which distinguished between the young and the elderly (R2 = 0.794, P < 0.05 for 1000 permutations) (Figure 6A). The VIP score analysis showed that four steroid metabolites contributed to the distinction between the young and the elderly, of which only DHEA-S levels are significantly lower in the elderly (Figure 6B and C).

## 4 Discussion

Evidence suggests that environmental cues such as aging induce functional and structural alterations in the adrenal cortex [50]. A previous report has shown marked changes in gene expression in the mouse adrenal gland with aging [51]. However, given the marked species differences in adrenal function and structure [9], the underlying mechanism of human adrenal aging is poorly understood. Here, we conducted bulk RNA-seq, scRNA-seq, and ST analyses of the aged human adrenal cortex. This study is the first detailed analysis of the aged human adrenal cortex at single-cell resolution and helps address how human adrenal aging develops, thereby leading to a better understanding of the pathophysiology of age-related disorders associated with adrenal aging.

The data from this study demonstrate that the inflammatory response and cellular senescence increase in the adrenal cortex with aging, which is most pronounced in ZF. Importantly, GRN and *in silico* perturbation analyses suggested that AP-1, which are activated in the aged human adrenal cortex, induce gene expression changes in ZF cells of the elderly, thereby inhibiting the differentiation from ZF to ZR cells. With aging, the adrenal cortex may be exposed to more pituitary-derived ACTH because of decreased sensitivity to cortisol feedback inhibition for ACTH secretion, thereby promoting adrenocortical cell proliferation and steroidogenesis [[52], [53], [54]]. Given that AP-1 are activated in the rat adrenal cortex in response to ACTH [55,56], it is conceivable that AP-1 are activated in the human adrenal cortex because of the age-related activation of the hypothalamic–pituitary–adrenal axis. Because ZF cells have shorter telomere length than ZG and ZR cells [57], ZF cells would be more prone to cellular senescence. Taken together, these observations suggest that AP-1 induce cellular inflammation when activated, thereby rendering ZF cells susceptible to cellular senescence. On the other hand, the expression of genes associated with WNT/β-catenin signaling is downregulated in ZG cells of the elderly, and knockout of *LEF1*, a major target gene for WNT/β-catenin signaling, promotes the differentiation of ZG to ZF cells. These observations are consistent with those of a previous rodent experiment showing that WNT/β-catenin signaling is required for the maintenance of ZG [41,[58], [59], [60]]. Therefore, it is likely that both the activation of AP-1 in ZF and attenuation of WNT/β-catenin signaling in ZG inhibit centripetal differentiation from ZG through ZF to ZR cells, thereby contributing to the expansion of ZF with a reciprocal reduction of ZG and ZR in the elderly. Although ZR-like cells occur centripetally from the ZF in genetically engineered mice with enhanced cAMP/PKA signaling [10,12], there was no significant difference in the expression of genes associated with cAMP/PKA signaling between the young and elderly in this study. Further studies are required to elucidate the involvement of cAMP/PKA signaling in human adrenal aging.

Evidence suggests that the continuity of aldosterone-secreting cells in the ZG is disrupted in aged human adrenocortical tissues, where aldosterone-producing micronodules (APMs) are found scattered as clonal cell clusters with autonomous aldosterone secretory capacity [1]. In this study, the area, cell number, and continuity of the ZG were reduced with aging, which might be caused by the attenuation of WNT/β-catenin signaling in ZG cells of the elderly. These observations are consistent with the immunohistochemical analysis of aged human adrenocortical tissues [2] and a phenotypic analysis of genetically engineered mice with altered WNT/β-catenin signaling [61]. Interestingly, *CYP11B2* expression was elevated in ZG cells of the elderly. Since a subset of ZG cells is transformed into APMs [13], the increased expression of *CYP11B2* in ZG cells of the elderly might play a role in the transition from ZG cells to APMs. Notably, the expression of *CYP11B1*, *CYP21A2*, and *HSD3B2* increased, whereas that of *CYB5A* decreased in ZR cells, and that of *CYP17A1* and *CYP11B1* increased in ZG cells. The bias of steroidogenesis toward cortisol synthesis in both ZR and ZG cells represents the age-related plasticity of adrenocortical steroid-producing cells, which, in addition to the expansion of ZF *per se*, might contribute to the increased production of cortisol. Recently, Valle et al. integrated fetal adrenal scRNA-seq/ST analysis and reported steroid synthesis in the fetal adrenal cortex during development [62]. Steroid synthesis in the adrenal cortex in both adults and fetuses was qualitatively stage-dependent. In particular, fetal and aged adrenal glands share a shift toward cortisol synthesis, and the changes in the fetal adrenal gland may provide clues to elucidate the mechanisms of age-related changes. Further studies are required to elucidate the mechanisms by which layer-specific steroidogenesis is impaired with aging.

In this study, we found that with aging, the circadian rhythm of cortisol is impaired, with marked alterations in the plasma steroid profile, such as a decrease in the adrenal androgen DHEA-S level, which might be due at least partly to the expansion of ZF and reduction of ZR. Abnormal cortisol production is associated with age-related disorders such as dementia, sarcopenia, and metabolic abnormalities [5,63,64]. Furthermore, age-related changes in the ZR have been noted as potential mediators of the aging process, as reduced adrenal androgens are associated with a variety of age-related disorders [6,65,66]. On the other hand, a couple of previous reports have shown that disruption of the ZG is involved in the development of salt-sensitive hypertension and primary aldosteronism [1,67], both of which increase with aging. Thus, the modulation of age-related alterations in multiple signaling pathways may offer a novel therapeutic strategy to prevent or treat age-related disorders arising from adrenal aging.

To the best of our knowledge, no detailed studies have investigated the pathophysiological role of macrophages even though they are a major immune cell subset in the human adrenal cortex [8,45,68,69]. In this study, we found that the proportion of macrophages increased in the ZF of the elderly, where the phagocytic activity of macrophages seemed to be enhanced. With aging, ZF cells, once they cease to proliferate and differentiate, might undergo cellular inflammation and senescence, and thereafter, being cleared by macrophages. However, when the number of senescent ZF cells exceeds the phagocytic capacity of macrophages, they may accumulate and even die, thereby accelerating age-related structural and functional alterations in the human adrenal cortex. On the other hand, the data from this study suggest that macrophages of the elderly have increased cholesterol metabolism. Recently, Conan et al. reported that cholesterol-containing macrophages are involved in steroid hormone synthesis in mouse adrenal cortex and that these macrophages increase with aging [70]. It is, therefore, interesting to speculate that macrophages as marked by “cholesterol homeostasis,” when they are increased in aged human adrenal cortex, particularly in ZF, respond to the increased cholesterol demand for steroid hormone synthesis. Our data provide clues for understanding how and which subtypes of macrophages are involved in the process of human adrenal aging.

This study has several limitations. Firstly, we used a small sample size for scRNA-seq and ST analysis, with a total of three cases; one young and two elderly. The structural alterations observed in our ST analysis were consistent with those reported in a previous immunohistochemical study [1,2]. Furthermore, using the GTEx RNA-seq data with a sufficient number of samples, we confirmed that the scRNA-seq and ST data obtained in this study were roughly comparable with the GTEx data in terms of steroidogenic gene expression. Second, the samples used for scRNA-seq and ST analyses were from a young female and two elderly males. Given the presence of sex differences in various adrenocortical diseases [71,72], we examined gene expression changes using GTEx data between young males and elderly females or young females and elderly males, and confirmed that the changes in inflammation- and senescence-associated gene expression are consistent with the results of scRNA-seq/ST analyses. Nonetheless, further studies with larger sample sizes for both males and females are required to validate the underlying mechanisms of human adrenal aging. Third, the adrenal glands associated with pheochromocytoma and non-functioning adrenal tumors were used for scRNA-seq and ST analyses. The possibility that these tumors alter gene expression in adrenocortical cells cannot be ruled out. Although scRNA-seq was performed on the samples from which the tumor areas were removed, the possible contamination of tumor cells cannot be excluded. Fourth, the samples used for the plasma steroid profiles were from patients with non-functioning adrenal tumors. The possibility that the plasma steroid profile was affected by potential steroid synthesis from the tumor cannot be ruled out. Fifth, the resolution of the ST analysis is limited; one spot is 55 μm in diameter and is estimated to contain 5–10 cells. The discrepancy between scRNA-seq and ST analysis in the change in ZR proportions with aging may be due, at least in part, to spots containing both ZF and ZR cells at the layer boundaries being classified as ZR.

**Figure 7:**
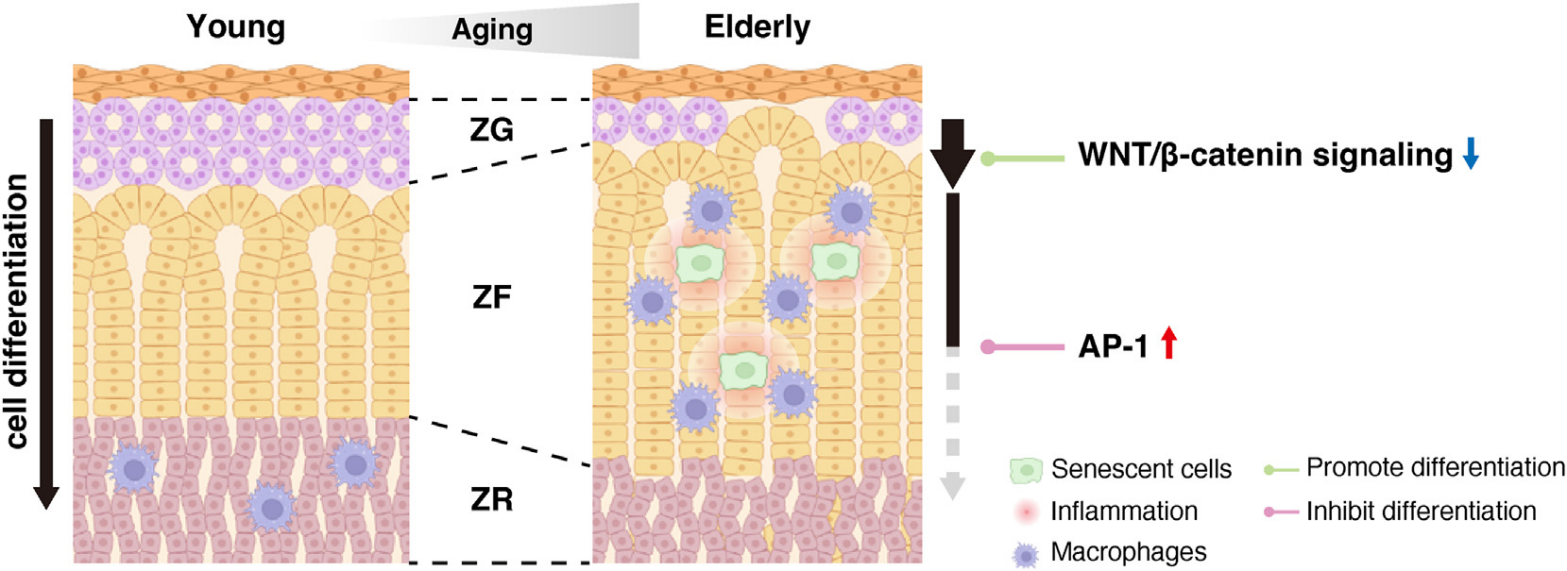
**Graphical abstract**. The human adrenal cortex undergoes the unique functional and structural alteration with aging. Our data suggest that both activation of the AP-1 in ZF and attenuation of WNT/β-catenin signaling in ZG inhibit the centripetal differentiation from ZG through ZF to ZR cells, thereby contributing to the expansion of ZF with reciprocal reduction of ZG and ZR in the elderly. Once, ZF cells cease proliferation and differentiation, might undergo cellular inflammation and senescence, which are phagocytosed by macrophages. However, when such senescent ZF cells exceed the phagocytic capacity of macrophages, they might accumulate and even die, thereby accelerating the process of human adrenal aging.

In conclusion, this study provides evidence that the layer-specific alterations in multiple signaling pathways underlie the abnormal layered structure and layer-specific changes in steroidogenic cells in the adrenal cortex with aging (Figure 7). Macrophages are also likely to contribute to age-related functional and structural alterations in the human adrenal cortex. Our data suggest that delaying the adrenal aging process offers a novel therapeutic strategy for preventing or treatment of age-related metabolic disorders associated with adrenal aging.

## CRediT authorship contribution statement

**Norifusa Iwahashi:** Writing – original draft, Visualization, Formal analysis, Data curation, Conceptualization. **Hironobu Umakoshi:** Writing – review & editing. **Masamichi Fujita:** Writing – review & editing. **Tazuru Fukumoto:** Writing – review & editing. **Tatsuki Ogasawara:** Writing – review & editing. **Maki Yokomoto-Umakoshi:** Writing – review & editing. **Hiroki Kaneko:** Writing – review & editing. **Hiroshi Nakao:** Writing – review & editing. **Namiko Kawamura:** Writing – review & editing. **Naohiro Uchida:** Writing – review & editing. **Yayoi Matsuda:** Writing – review & editing. **Ryuichi Sakamoto:** Writing – review & editing. **Masahide Seki:** Methodology. **Yutaka Suzuki:** Methodology. **Kohta Nakatani:** Methodology. **Yoshihiro Izumi:** Methodology. **Takeshi Bamba:** Methodology. **Yoshinao Oda:** Methodology. **Yoshihiro Ogawa:** Writing – review & editing, Project administration.

## Declaration of competing interest

The authors declare that they have no known competing financial interests or personal relationships that could have appeared to influence the work reported in this paper.

## Data Availability

The codes are archived at Zenodo (https://zenodo.org/doi/10.5281/zenodo.10230663). The scRNA-seq and ST fastq files and count data are available on ArrayExpress (accession number E-MTAB-13615 and 13991).
