# Supplementary material for: Single-cell and spatial transcriptomics analysis of human adrenal aging
Source: Mol Metab. 2024 May 6;84:101954. doi: 10.1016/j.molmet.2024.101954 (PMC11101872; doi:10.1016/j.molmet.2024.101954)
Supplement: Multimedia component 2 [file mmc2.pdf]

Supplementary Information

Single-Cell and Spatial Transcriptomics Analysis of Human Adrenal Aging

Norifusa Iwahashi, Hironobu Umakoshi, Masamichi Fujita, Tazuru Fukumoto, Tatsuki Ogasawara, Maki Yokomoto-Umakoshi, Hiroki Kaneko, Hiroshi Nakao, Namiko Kawamura, Naohiro Uchida, Yayoi Matsuda, Ryuichi Sakamoto, Masahide Seki, Yutaka Suzuki, Kohta Nakatani, Yoshihiro Izumi, Takeshi Bamba, Yoshinao Oda, Yoshihiro Ogawa

<u>Contents:</u>

Supplementary Fig. 1–8

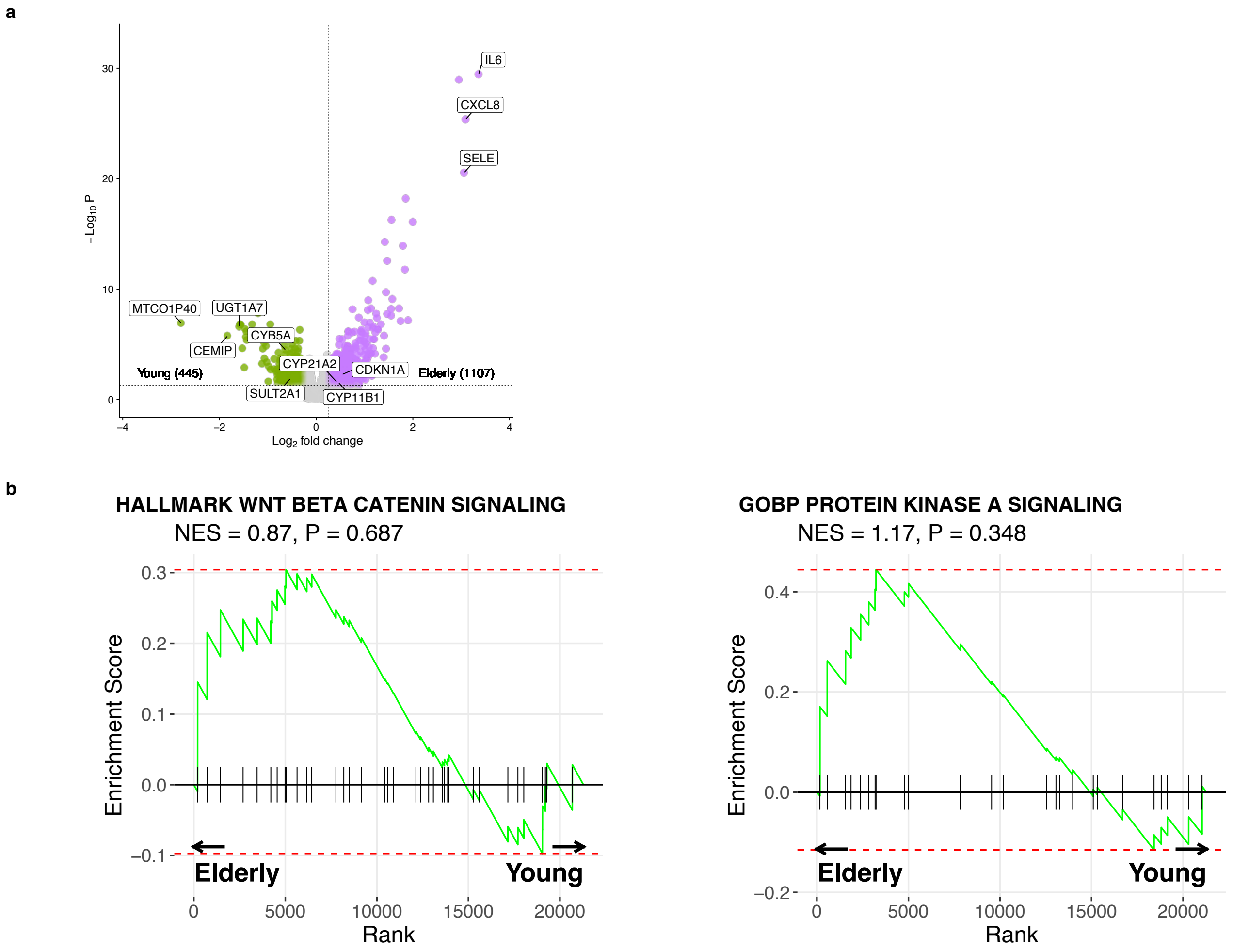

Supplementary Fig. 1: Differential expression analysis and gene set enrichment analysis (GSEA) of GTEx data.

a Volcano plot showing the results of differential expression analysis of GTEx data between the young and elderly. Genes with positive log2 fold change are upregulated in the elderly. Genes with top3 log2 fold change and steroidogenic genes with significant difference are labeled. **b** GSEA results for WNT/β-catenin and protein kinase A (PKA) signaling gene sets. Hallmerk WNT/β-catenin signaling and gene ontology biological process (GOBP) protein kinase A signaling gene sets are shown. Gene sets with positive normalized enrichment scores (NES) represent positive enrichment in the elderly.

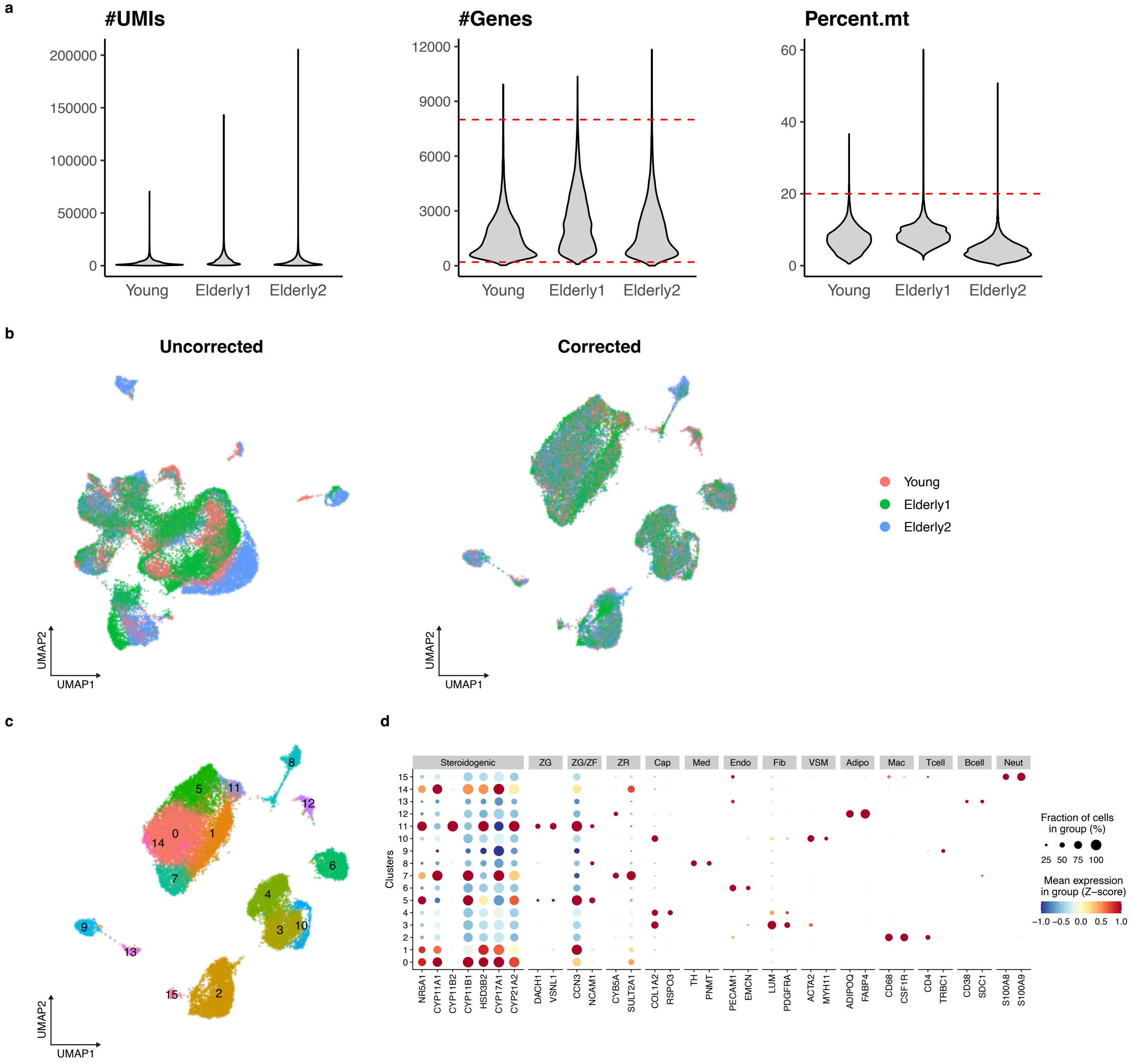

Supplementary Fig. 2: Quality control, batch correction, and clustering of scRNA-seq data.

a Violin plots showing quality control metrics of scRNA-seq data. (Left panel) number of unique molecular identifiers (UMI) per cell, (center panel) number of expressed genes per cell, (right panel) percentage of mitochondrial genes per cell. The red horizontal dotted lines in the panels represent the quality control thresholds. The upper line is 8,000 and lower line is 500 in (center panel), and the line in (right panel) is 20. **b** UMAP plots of scRNA-seq data before and after batch effect correction, colored by sample. (Left panel) before batch correction, (right panel) after batch correction. **c** UMAP plot of scRNA-seq data colored by the result of unsupervised clustering. **d** Dot plot showing the expression of marker genes in unsupervised clusters of scRNA-seq data.

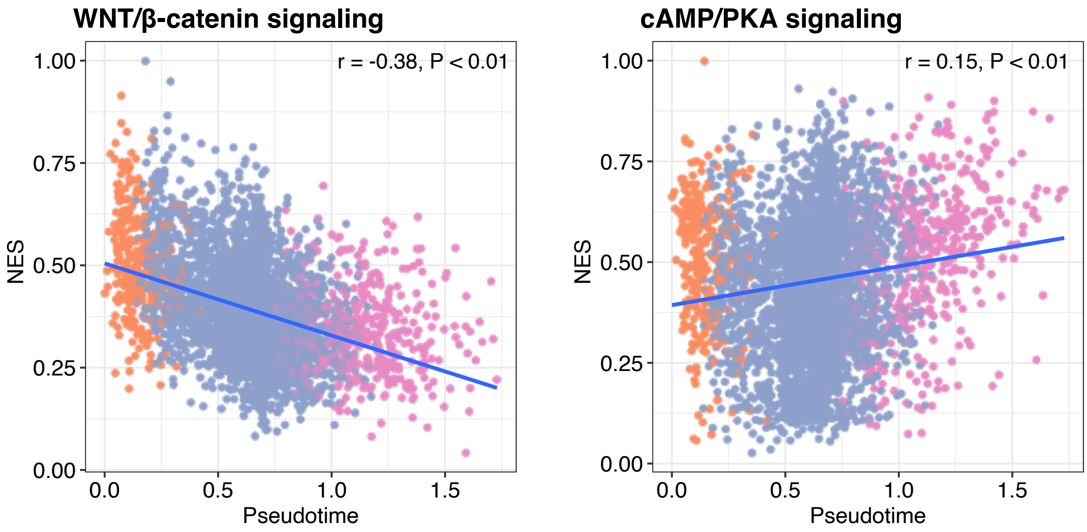

Supplementary Fig. 3: Changes in WNT/β-catenin and cAMP/PKA signaling along the adrenocortical cell differentiation trajectory. Dot plot showing the correlation between the pseudotime and the normalized enrichment score (NES). Dot colors are annotated cell types, blue line is the linear regression line.

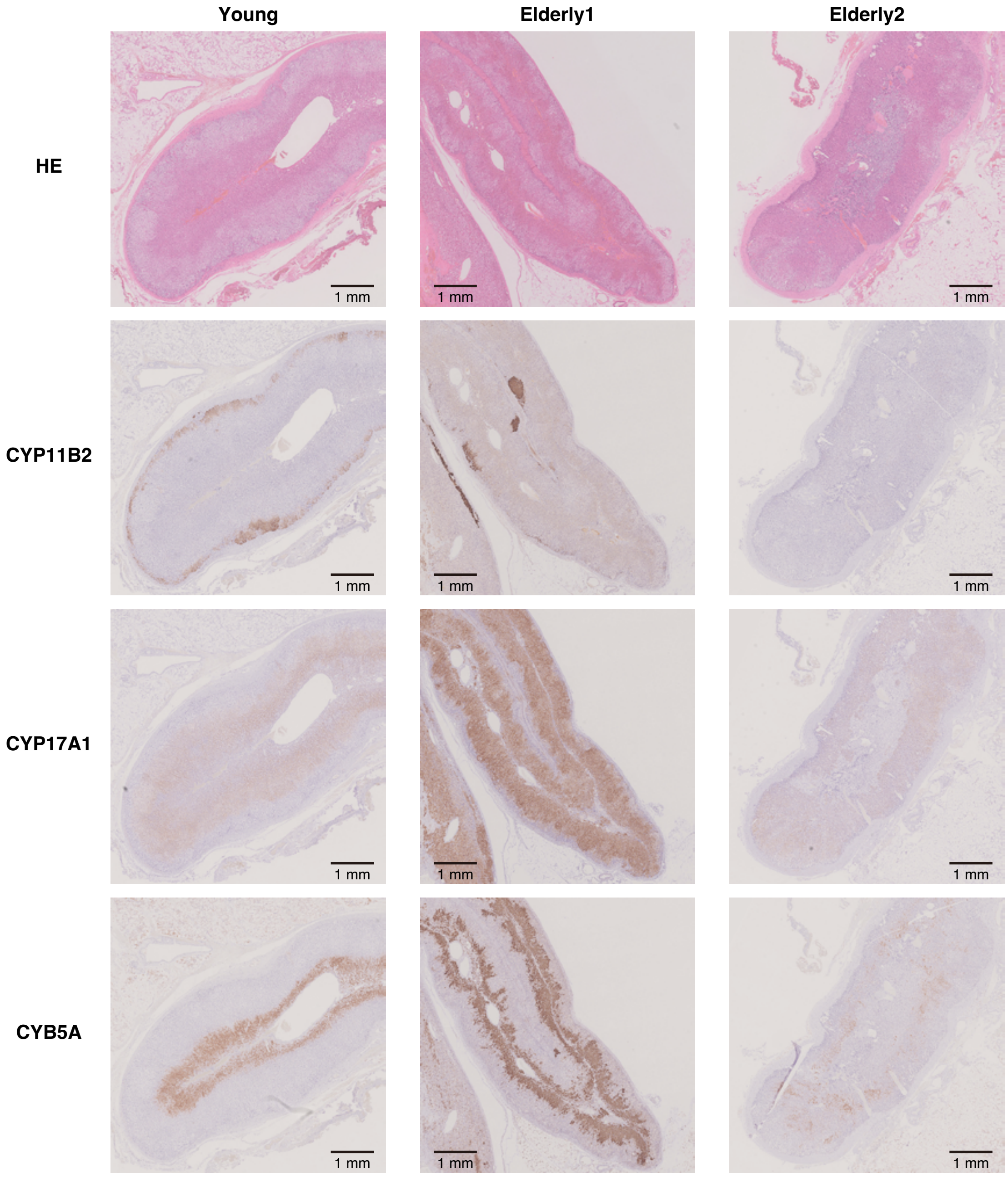

Supplementary Fig. 4: Immunohistochemical staining of each sample.

Immunohistochemical staining of samples used for scRNA-seq and ST. Young in the left column, Elderly1 in the middle column, and Elderly2 in the right column. From top row to bottom: hematoxylin-eosin, CYP11B2, CYP17A1, CYB5A. The black bar in the images is 1 mm.

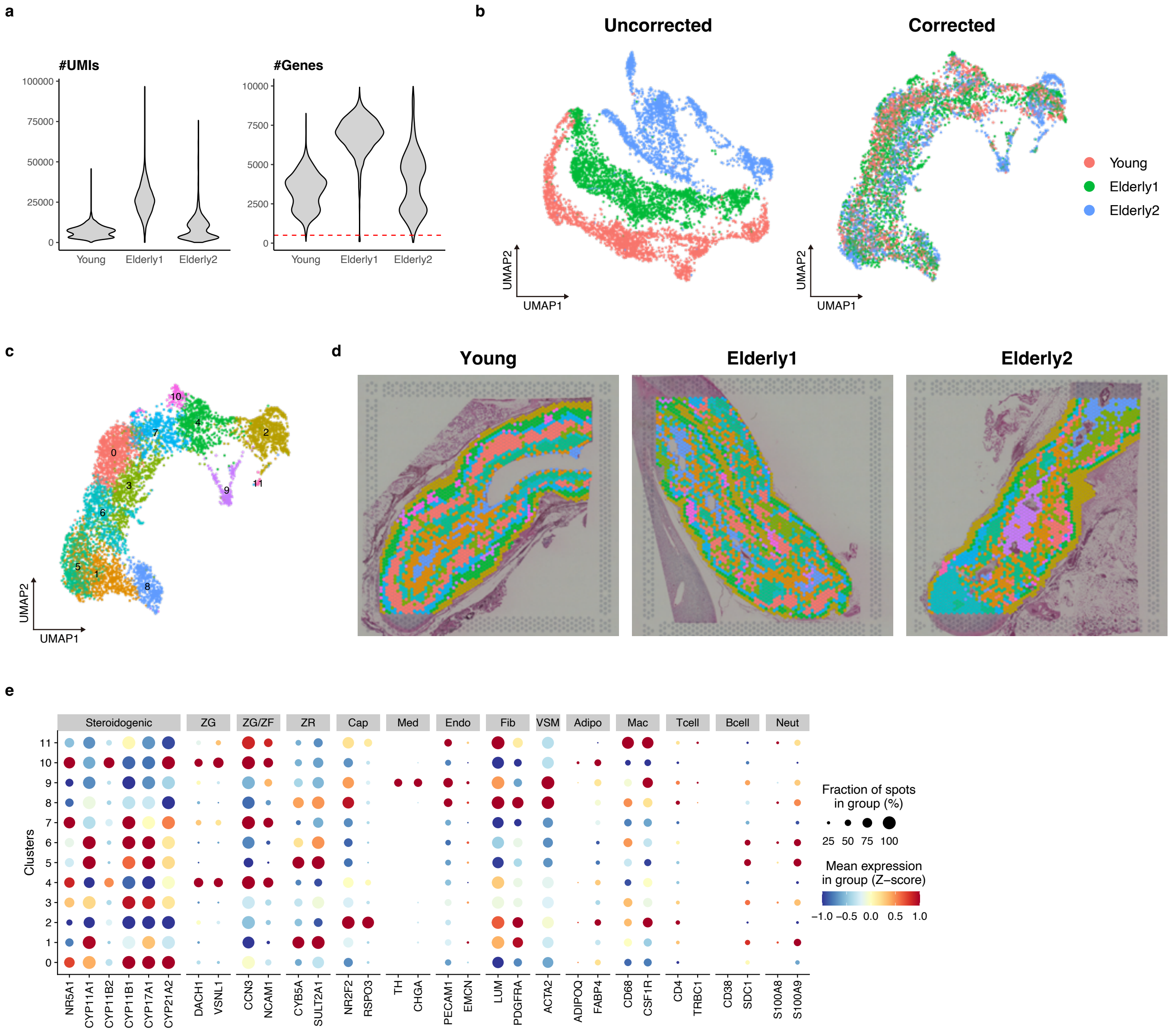

Supplementary Fig. 5: Quality control, batch correction, and clustering of ST data.

a Violin plots showing quality control metrics of ST data. (Left panel) number of unique molecular identifiers per spot, (right panel) number of expressed genes per spot. The red horizontal dotted line in (right panel) represents the quality control threshold, which is 500. **b** UMAP plots of ST data before and after batch effect correction, colored by sample. (Left panel) before batch correction, (right panel) after batch correction. **c** UMAP plot of ST data colored by the result of unsupervised clustering. **d** Plot showing clustering results and cluster locations of ST data. **e** Dot plot showing the expression of marker genes in unsupervised clusters of ST data.

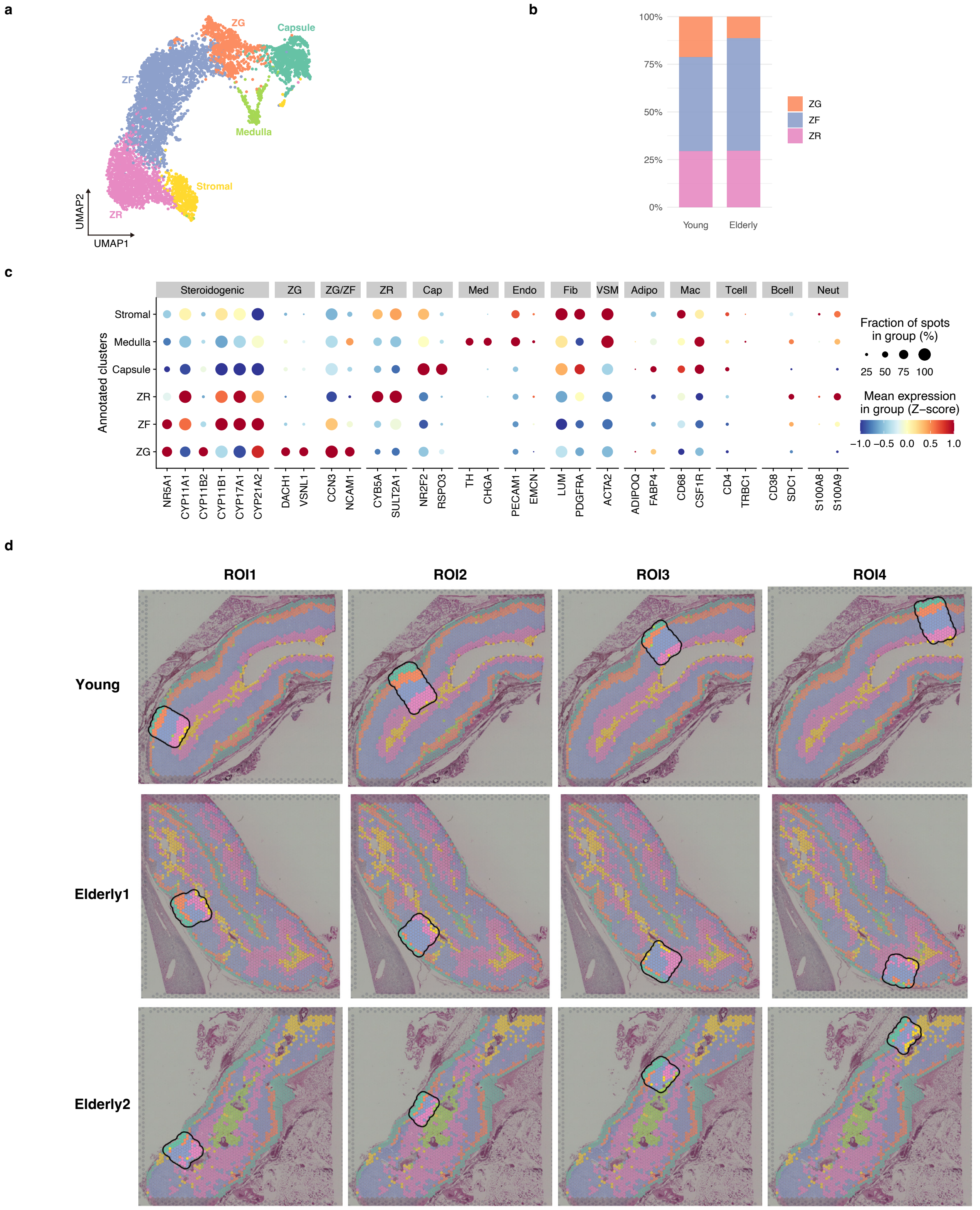

Supplementary Fig. 6: ST data after annotation.

a UMAP plot of ST data colored by cell type annotation. **b** Bar plot showing the composition of ST spots annotated with ZG, ZF, and ZR in the young and elderly in the ROI shown in d. **c** Dot plot showing the expression of marker genes in annotated clusters of ST data. **d** Four ROIs of each sample in assortativity analysis.

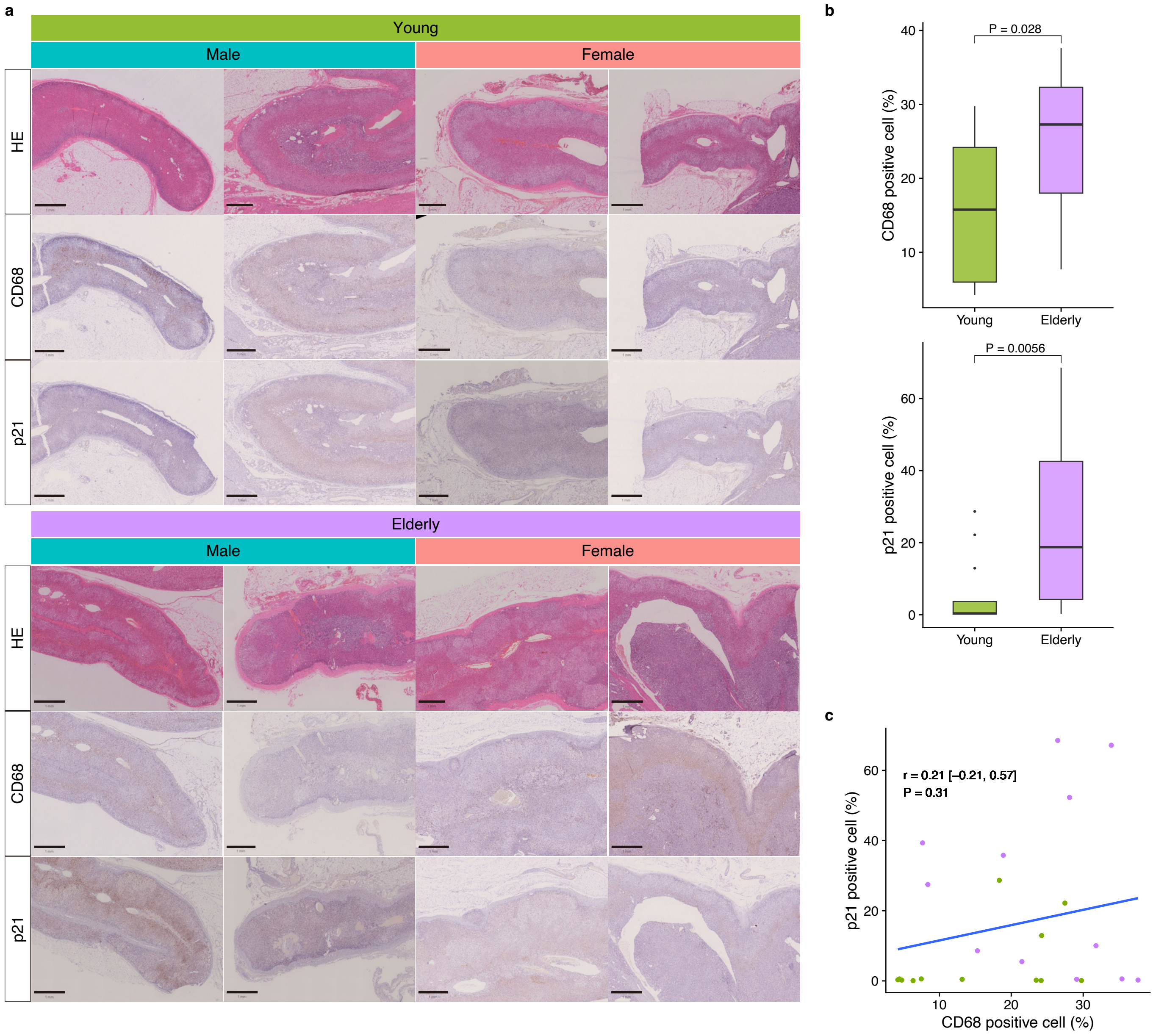

Supplementary Fig. 7: Immunohistochemical (IHC) analysis of adrenal tissues adjacent to pheochromocytoma or non-functioning cortical tumor.

a IHC of hematoxylin-eosin, CD68, and p21 in eight adrenal tissues. The black bar in the images is 1 mm. The upper panel shows, from left to right, cases 1-4 (young group) and the bottom panel shows cases 5-8 (elderly group). Detailed patient characteristics are provided in Supplementary Table 17. **b** Box plot showing IHC analysis results. The percentage of positively stained cells was compared between the young and elderly (upper panel: CD68; bottom panel: p21). The P-values of the Wilcoxon rank sum test are shown. **c** Dot plot showing the correlation between the percentage of CD68 and p21-positive cells. The blue line is the regression line based on the least-squares method. The results of the Pearson correlation analysis are displayed in the upper left corner.

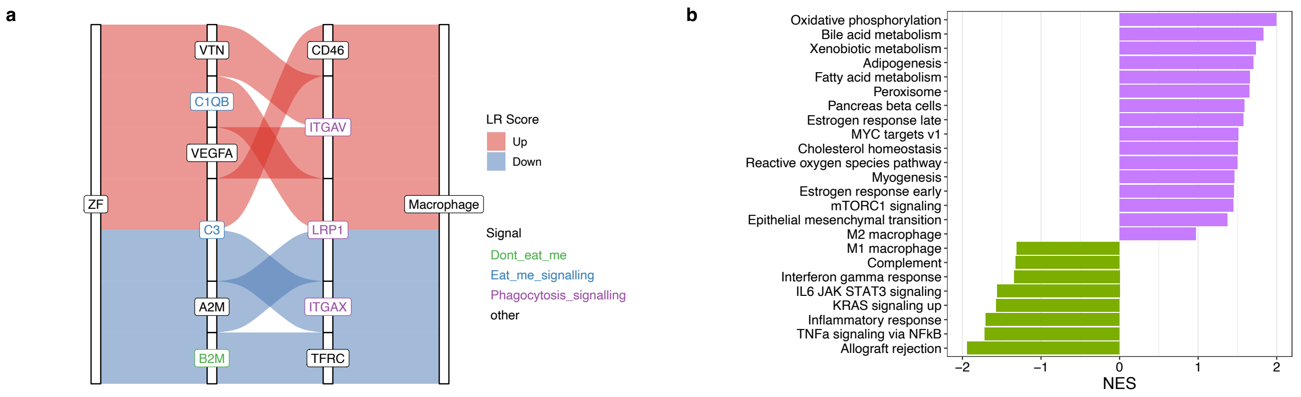

Supplementary Fig. 8: Characterization of macrophages of the elderly.

a Sankey plot showing the result of cell-cell communication analysis. The interactions with ZF cells as the source and macrophages as the target, which are related to phagocytosis (gene list in Supplementary Table 18), are shown. Interactions with higher LR scores for the elderly than for the young are colored red and those with lower LR scores are colored blue. **b** Bar plot showing the results of the gene set enrichment analysis comparing macrophages of the young and elderly. Significantly enriched or M1/M2 macrophage gene sets are shown. The gene set with a positive normalized enrichment score (NES) indicates the enrichment of genes whose expression was upregulated in the elderly.
